# Exploring Life Detection on Mars: Understanding Challenges in DNA Amplification in Martian Regolith Analogue After Fe Ion Irradiation

**DOI:** 10.3390/life15050716

**Published:** 2025-04-29

**Authors:** Alessia Cassaro, Claudia Pacelli, Silvano Onofri

**Affiliations:** 1 Science and Innovation Directorate, Italian Space Agency, via del Politecnico, 00133 Rome, Italy; alessia.cassaro@asi.it; 2 Department of Ecological and Biological Sciences, University of Tuscia, Largo dell’Università snc, 01100 Viterbo, Italy; onofri@unitus.it

**Keywords:** biosignatures, building blocks, space exploration, gene amplification, Mars Sample Return

## Abstract

The search for life beyond Earth currently hinges on the detection of biosignatures that are indicative of current or past life, with terrestrial life being the sole known example. Deoxyribonucleic acid (DNA), which acts as the long-term storage of genetic information in all known organisms, is considered a biosignature of life. Techniques like the Polymerase Chain Reaction (PCR) are particularly useful as they allow for the amplification of DNA fragments, allowing the detection of even trace amounts of genetic material. This study aimed to detect DNA extracted from colonies of an Antarctic black fungus both when (i) alone and (ii) mixed with a Sulfatic Mars Regolith Simulant (S-MRS), after exposure to increasing doses of Fe ions (up to 1 kGy). PCR-based amplification methods were used for detection. The findings of this study revealed no DNA amplification in samples mixed with Sulfatic Mars Regolith Simulant, providing important insights into the potential application of these techniques for in situ DNA detection during future space exploration missions or for their application on the Mars sample return program; it also gives input in the planetary protection discussions.

## 1. Introduction

With the landing of the Perseverance rover in the Jezero Crater in February 2021, the upcoming launch of the Rosalind Franklin rover, and the subsequent Mars Sample Return (MSR) campaign, the search for traces of life on Mars is becoming increasingly tangible [1]. The search for life beyond Earth primarily focuses on detecting biosignatures of extant or recently extinct life. A biosignature is defined as an object, substance, and/or pattern created by a biological source [2,3]. Biosignatures encompass various indicators of past life, including heteroatoms within graphitic carbon and isotopic variations between reduced carbon and carbonates in ancient rocks [4]. They also include molecular biomarkers or their fragments [5,6], fossil-like cellular structures [7], and potential biogenic formations found in diagenetic concretions, granules, and rosettes [8]. Additionally, biosignatures may manifest as microbially influenced structures, such as stromatolite-like morphologies [9,10] and microbially induced sedimentary structures (MISS; [11]). Their study depends on sophisticated experimental studies, as many different signatures of life can be used [12]. Among these, biomolecules such as nucleic acids, which encode the genetic information of organisms, are essential components of life and hold significant potential as biosignatures. DNA is an excellent biological marker for several reasons:

(i) it is present in all known forms of life and serves as a highly specific indicator of biological activity [13];
(ii) it demonstrates remarkable stability when adsorbed onto mineral surfaces;
(iii) the information it carries is resilient to degradation
(iv) the formation of its double-stranded, polymeric structure under purely abiotic conditions is highly improbable [14,15].

Among biomarkers, nucleic acids are an excellent example of biotic biosignatures, a feature or measurement that exists only if biology generated it or it was undoubtedly modified by life [16]. Although we do not yet know how life on Mars might have originated, if it ever existed, it is reasonable to base our studies on the only example of life we have: terrestrial life [17]. This does not automatically imply that putative extraterrestrial DNA would be an exact replica of the terrestrial counterpart, but it is possible that life beyond Earth utilizes different molecules to store genetic information (e.g., peptide nucleic acids; [18]). Nevertheless, the existence of DNA-like molecules in extraterrestrial life may be plausible due to frequent meteoritic exchanges between planetary bodies. The building blocks of life have been delivered to Earth from space: nucleobases such as guanine, hypoxanthine, xanthine, adenine, and uracil have been identified in carbonaceous chondritic meteorites [19,20], while riboses have been detected in the gaseous phases of the interstellar medium [21]. This raises a reasonable possibility that potential past or present life on Mars could share similarities with life on Earth [22]. However, it must be noted that DNA molecules in extraterrestrial environments, such as Mars or outer space, are exposed to stressors like vacuum or low-pressure conditions and high levels of radiation [23]. The present-day conditions on Mars, including its cold and dry climate, atmospheric composition (H₂O, CO₂, N₂, and noble gases), low atmospheric pressure (6–11 mbar), meteorite impacts, and radiation environment (Galactic Cosmic Rays dose of 232 mGy/y), could interfere with the preservation of biological signatures of life [24,25,26,27,28].

These factors can have harmful effects on DNA, given its relative instability compared to other biomolecules like lipids and amino acids [29,30]. While no current rovers are equipped with instruments capable of detecting nucleic acids, several experimental approaches have been developed to support the search for DNA on Mars. Ref. [31] designed the Search for Extra-Terrestrial Genomes (SETG) instrument for in situ extraction and nanopore sequencing of nucleic acids from Mars analogue samples collected during field campaigns. Similarly, the Oxford Nanopore Technologies (ONT) MinION sequencer has demonstrated the capability to detect DNA at concentrations as low as ~0.001 ng [32]. Advanced experiments have shown that the MinION sequencer can reliably detect and identify species with as little as 2 pg of DNA and analyze microbiomes with 600 pg of DNA extracted from 500 mg of soil samples [33]. The MinION has demonstrated the ability to detect not only nucleic acids but also their derivatives containing nonstandard bases, such as inosine [34], as well as potentially xeno-NAs (XNAs) and other informational polymers [35]. Additionally, previous studies have demonstrated that the reagents required for DNA detection and MinION sequencing can withstand radiation doses comparable to those expected during a mission to Mars [36]. Ref. [37] reported the effectiveness of pre-existing automated and semi-automated extraction systems, utilizing different cell lysis and purification methods, for DNA extraction from different analogue environments in the Canadian High Arctic (soils, cryoconites, subzero saline spring sediments, and cryptoendoliths), followed by detection through MinION sequencing.

In the search for life on other planets, DNA can serve as a detectable biosignature, not only within isolated living organisms but also as evidence of past life in the form of preserved cells or genetic material. On Earth, ancient DNA has been successfully extracted from a variety of materials, including permafrost, amber, and salt crystals [38,39,40]. However, the timescales over which DNA can persist remain a topic of debate. Certain environmental conditions on Earth, and potentially on Mars, could favor the long-term preservation of DNA. Factors such as halite crystals, low temperatures, and arid conditions are known to contribute to DNA stability [41,42]. For example, ref. [43] documented the survival of bacteria in frozen environments, raising intriguing possibilities for DNA preservation and recovery from the Martian subsurface environment. Ref. [44] suggested that nucleic acid-based life detection on Mars may be feasible in scenarios involving extant or recently deceased life with intact cells. However, extracellular DNA can degrade upon hydration or bind irreversibly to minerals, making its detection more challenging. To enable effective nucleic acid detection, it is essential to understand the specific conditions under which DNA interacts with minerals. Research has shown that clays, for instance, can adsorb over 99% of DNA, making recovery nearly impossible [45]. Similarly, other silicates and non-silicates exhibit very low DNA recovery rates (<1%). In contrast, quartz and iron oxides show relatively lower DNA adsorption (<50%), which is encouraging, as iron oxides are particularly significant in the search for life on Mars [46]. Recent advances by [47] have led to the development of highly sensitive methods for detecting and quantifying trace amounts of DNA from the JSC-1 Martian regolith simulant. As the Mars Sample Return (MSR) mission aims to bring back samples from the Martian surface by 2033, understanding how DNA is preserved in different mineral matrices and refining nucleic acid extraction techniques are crucial. These samples, consisting primarily of rocks and regolith collected by the Perseverance rover from the top 5 cm of the Martian surface, hold significant potential for deepening our knowledge on potential life on Mars [48]. In this context, the purpose of this study is to evaluate whether DNA can be protected by Sulfate Martian Regolith Simulant (S-MRS) under a simulated radiation environment, in the context of Martian exploration. Specifically, we tested the resistance of fungal DNA, both alone and mixed with Sulfate Martian Regolith Simulant (S-MRS), after exposure to increasing doses of accelerated Fe ions (up to 1000 Gy). It should be highlighted that our highest dose of 1000 Gy corresponds to approximately 13.200 years on Martian surface (extrapolated from [28] which reported the dose of 76 mGy/yr), a timeframe considered suitable for conducting ground-based simulation studies.

The experiments assessed the ability of DNA to be amplified using common molecular biology techniques, including Polymerase Chain Reaction (PCR) for short DNA fragments, Random Amplified Polymorphic DNA (RAPD) for whole-genome analysis, and quantitative Polymerase Chain Reaction (qPCR). The findings of the study also have implications for the theory of lithopanspermia, the hypothesis that life can be spread among planets and planetary systems through rock fragments [49].

## 2. Materials and Methods

### 2.1. Test Organism

The black fungus *Cryomyces antarcticus* MNA-CCFEE 515, a cryptoendolithic species from Antarctica, was originally isolated from sandstone samples at Linnaeus Terrace in the McMurdo Dry Valleys (Southern Victoria Land, Antarctica) by H. Vishniac during the 1980–1981 Antarctic expedition. This fungus was previously selected as a test organism due to its polyextremophilic ability [50]. For this experiment, the fungus was cultivated on Malt Extract Agar (MEA), composed of 30 g/L malt extract, 5 g/L peptone, and 15 g/L agar (AppliChem GmbH, Darmstadt, Germany), and incubated under laboratory conditions at 15 °C for three months. Prior to irradiation experiments, micro-colonies were collected from Petri dishes, transferred into sterile 2 mL tubes, and subsequently used for DNA extraction.

### 2.2. DNA Extraction and Sample Preparation

DNA was extracted from fungal colonies, using the Nucleospin Plant kit (Macherey-Nagel, Düren, Germany) following the protocol optimized for black fungi [51].

The extracted DNA originates exclusively from the tested organism, with no external contamination. To ensure this, contamination control measures were applied, including the use of negative controls during DNA extraction and PCR amplification, and sterilized reagents. Additionally, sequencing data were carefully analyzed to rule out the presence of exogenous DNA. The obtained sequences are consistent with the expected genetic profile of the target organism, further confirming the absence of contamination.

Two sets of samples were prepared: (i) DNA alone (hereafter directly exposed DNA) and (ii) DNA mixed with Sulfatic Mars Regolith Simulant (S-MRS). The mineralogical composition of the Martian regolith analogue is as follows (%*wt*): 32% gabbro, 15% olivine, 3% quartz, 13% hematite, 7% goethite, and 30% gypsum [52]. Minerals were previously dry-sterilized (140 °C for 4 h).

An additional set of samples was kept under laboratory conditions and used as a control for the experiment.

### 2.3. Fe Ion Irradiation Exposure

**Figure 1:**
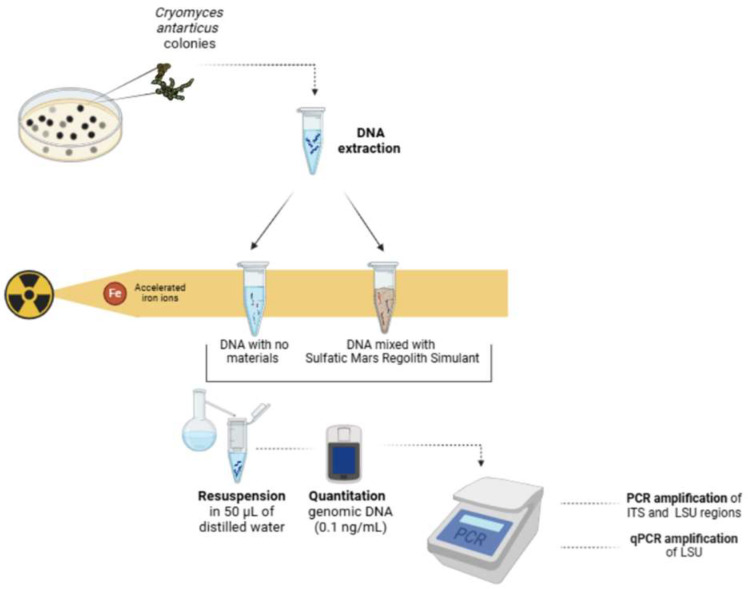
Flowchart of the experimental design. The DNA was extracted from *C. antarcticus* colonies and divided into two 1.5 mL Eppendorf tubes, one containing a Martian regolith analogue and the other without it. Both tubes were exposed to iron (Fe) ions to simulate environmental stress conditions similar to those experienced on Mars. After exposure, the extracted DNA was resuspended in nuclease-free water and quantified using spectrophotometry, achieving a final concentration of 0.1 ng/mL. Subsequently, the quantified DNA was subjected to amplification using two main techniques: PCR (Polymerase Chain Reaction) to produce multiple copies of the target DNA, and qPCR (quantitative PCR) to determine the exact amount of amplified DNA, monitoring the process in real time.

A total of 50 µL of the extracted fungal DNA alone and mixed with the S-MRS regolith analogue was placed within 200 μL PCR tubes, dried at 37 °C, and exposed to increasing doses of accelerated Fe ions (with an energy of 500 MeV/n (LET in water = 200 keV/μm)). Irradiations were performed at the Heavy Ion Medical Accelerator (HIMAC) facility of the National Institute for Radiological Sciences (NIRS) in Chiba, Japan. The applied doses were 50, 250, 500, and 1000 Gy and the dose rate was 12 Gy/min. Each sample was irradiated in triplicate and used for the following analyses. After irradiation, DNA was resuspended, adding 50 µL of distilled water. Quantitation of resuspended genomic DNA was performed using the Qubit system and all the samples were diluted to the same concentration (0.1 ng/mL). A schematic representation of the experimental setup and methodologies is shown in Figure 1.

### 2.4. Nucleic Acid Amplification and Estimation of DNA Lesions

Real-time PCR was used to quantify the total LSU fragments number. For the amplification of the target region, LR0R (ACCCGCTGAACTTAAGC) [53] and LR5 (TCCTGAGGGAAACTTCG) [54] primers were used. Amplifications were carried out in triplicate. The final reaction mixture consisted of 7.5 of μL of qPCR cocktail (iQ SYBR Green Supermix, Bio-Rad, MI, Italy) mixed with 1 μL of DNA template (0.1 ng/μL) and 1 μL of each primer for a final volume of 5 pmol/µL. Biorad CFX96 real-time PCR detection system (BioRad, Hercules, CA, USA) was programmed to operate at 94 °C for 5 min followed by another 45 sec at 94 °C, annealing at 52 °C for 30 min, and elongation at 72 °C for 2 min. Fluorescence measurements were recorded at the end of each annealing step. After 35 cycles, a melt curve analysis was performed by recording changes in fluorescence as a function of raising temperature from 60 to 95 °C in 0.5 °C-per-5 s increments. All data were carried out performing *n*  ≥  3 qPCR protocols, each one including *n* = 3 replicates. The relative number of DNA lesions in a 0.9 kb amplification product was calculated in samples exposed to different doses of Fe ions. Poisson distribution was calculated following the experiments in [55]: lesions/amplicon = −ln(At/Ao), where At represents the amplification of exposed samples and Ao the amplification of not-exposed controls.

### 2.5. Single-Gene PCR and Random Amplified Polymorphic DNA Analysis

Two overlapping tracts in the Internal Transcribed Spacer (ITS) regions and the Large SubUnit-coding Sequences (LSU) of the nuclear ribosomal RNA (rRNA) gene complex were amplified. The used primers were ITS4a (ATTTGAGCTGTTGCCGCTTCA) [56], ITS5 (GGAAGTAAAAGTCGTAACAAGG), and LR5 (TCCTGAGGGAAACTTC) [54]. PCR reactions were carried out for each sample in a solution consisting of 12.5 μL of BioMixTM (BioLine Ltd., London, UK), 1 µL of each primer solution (5 pmol/μL), and 0.2 ng of DNA template; nuclease-free water was added until the final volume of 25 µL was reached. MyCycler Thermal Cycler (Bio-Rad Laboratories GmbH, Munich, Germany) equipped with a heated lid was used and amplification conditions were as reported in [57]. The whole-genome integrity was assessed by Random Amplified Polymorphic DNA (RAPD). PCR reactions were carried out for each sample in a final solution containing 12.5 µL of BioMixTM, 5 pmol of primer (GGA)7 [58], and 0.2 ng of DNA sample, in a final volume of 25 µL. Amplifications were performed according to [57]. The quality of the amplified DNA fragments was analyzed by electrophoresis in a 1.5% agarose gel, performed in TAE buffer (40 mM Tris–acetate, 1 mM EDTA, pH 8.0) for 40 min at 100 V, stained with Gel RedTM Nucleic Acid Gel Stain. The agarose gel was visualized under UV light, at 260 nm with a trans-illuminator, and a digital image was recorded.

### 2.6. Evaluation of PCR Bands Intensity

To quantify the DNA amplification, intensity of the bands obtained by gel electrophoresis was analyzed by using Image J software 1.54k (NIH, Rasband (1997–2018)). All the gel images were converted in 8-bit format and the background was removed by subtracting the value of blank area. The intensity calculation was performed by selecting the band lane and plotting the gray values (the gray values of all the pixels in the selection divided by the number of pixels) against the distance (pixel). From the plotted graphs, the width of each peak was measured, and these values correspond to the light intensity. The values of light intensity obtained from each band were normalized with respect to the value of control samples.

## 3. Results

### 3.1. DNA Damage Assessment

#### Genomic DNA

After the irradiation exposure, DNA was resuspended from each sample (alone and mixed with S-MRS analogue) and was newly quantified. All the obtained amounts were reported in Table 1.

### 3.2. Nucleic Acid Amplification and Estimation of DNA Lesions

**Figure 2:**
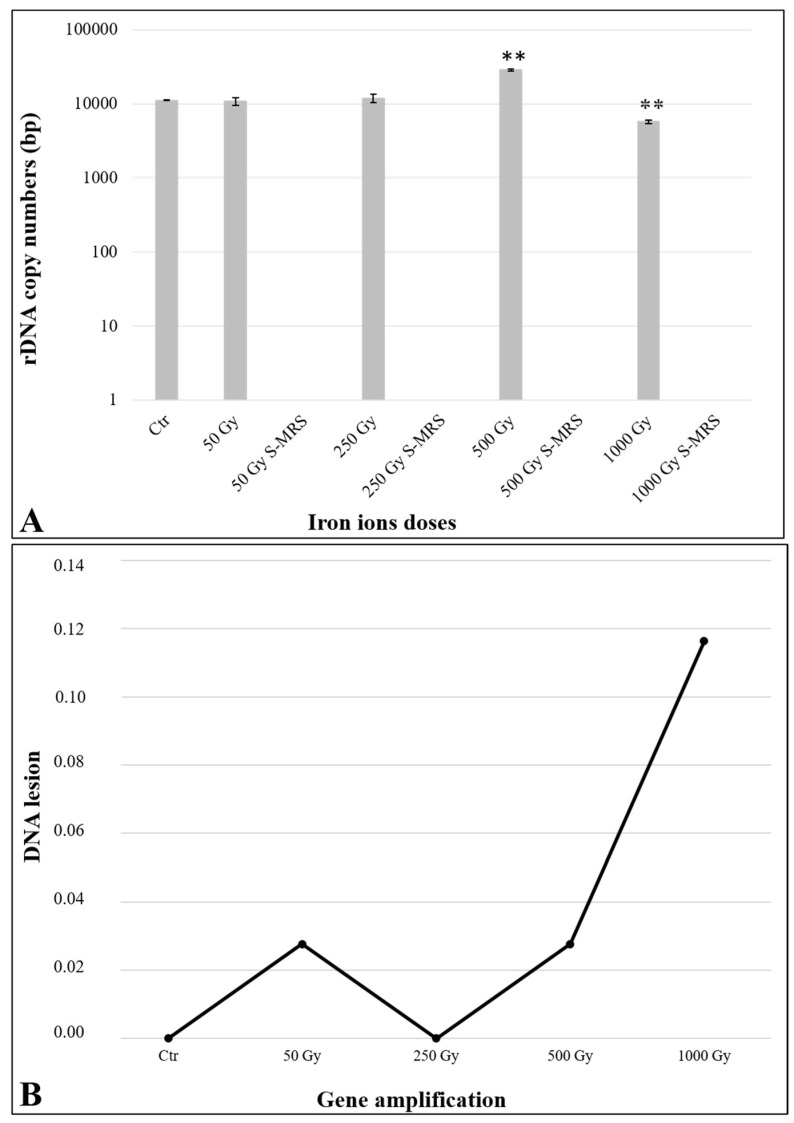
(**A**) Quantitative PCR of a 939 bp target gene (LSU) of *C. antarcticus* extracted DNA, exposed to increased doses of accelerated iron ions (500 MeV/n). The number of amplified copies on a logarithmic scale is shown on the *y*-axis, while treatments are expressed in kGy on the *x*-axis. (**B**) DNA lesions obtained after real-time PCR amplification of directly exposed DNA and DNA mixed with S-MRS after iron ion irradiation. Ctr: DNA extracted from *C. antarcticus* colonies not exposed to radiation treatment. Significant differences were calculated by *t* test with ** = *p* < 0.001.

Amplification of extracted DNA exposed to increasing doses of Fe ion irradiation was performed on a 939 bp-length gene, according to the protocol optimized in [55]. Figure 2A reports the quantity of amplicons detected on fungal DNA after exposure to increasing doses of Fe ions. Overall, amplification was obtained only for directly exposed DNA samples, with a slight decrease in the amplified copy numbers at the highest irradiation dose (1 kGy) (Figure 2A). No amplification was observed for the DNA samples mixed with S-MRS regolith analogue. Overall, an average of ~13,500 DNA copy numbers was detected (Figure 2A).

Real-time amplification values were used to estimate nucleic acid lesions of exposed samples in comparison with control samples. It should be noted that this evaluation was only possible for the directly exposed DNA samples, since no amplification was observed in the DNA samples mixed with S-MRS regolith analogue. Similar amounts of amplified DNA (Table 2, column 4) were detected in samples exposed to 50 and 250 Gy of Fe ions, when compared with control samples. As a result, a similar trend was obtained for the number of DNA lesions calculated by Poisson distribution on a ~0.9 kb fragment. Extended nucleic acid lesions were observed in samples exposed to the highest doses (500 and 1000 Gy) (Table 2, column 6). The estimated DNA damage for 0.9 kb gene is reported in Figure 2B.

### 3.3. DNA Integrity Assay by PCR Amplification and Bands Intensity

DNA integrity in two overlapping tracts in the ITS-LSU region with a length of 700 and 1600 bp within the rRNA gene complex was assessed by PCR amplification and gel electrophoresis assay. The aim of the test was to evaluate the presence of DNA Double-Strand Breaks (DSBs) after the irradiation with increasing doses of accelerated Fe ions with and without the presence of minerals. DNA amplification of the ITS region (700 bp) was observed in control and exposed samples for both sets of samples (directly exposed DNA and mixed with S-MRS analogue) (Figure S1A). On the contrary, the amplification of the 1600 bp gene (LSU region) did not occur for any of the irradiated DNA mixed with S-MRS regolith analogue (Figure S2A). Besides, the whole genome was investigated by an RAPD assay, which revealed preserved profiles for all the directly exposed DNA samples, even at the highest dose (1 kGy) (Figure S3A). All the DNA samples mixed with S-MRS regolith analogue lacked amplification bands (Figure S3A). Light band intensity was calculated to evaluate quantitative differences among samples. The light intensity results are in accordance with gel electrophoresis results: low-intensity values have been recorded for ITS amplification of DNA mixed with S-MRS analogue, compared with the amplification of directly exposed DNA (Figure S1B), while no intensity values were reported for the LSU amplification of DNA mixed with S-MRS Martian Regolith analogue (Figure S2B). The RAPD evaluation showed high light intensity values for directly exposed DNA and values close to zero for the amplification of DNA mixed with S-MRS Martian Regolith analogue (Figure S3B).

## 4. Discussion

The search for life on Mars has garnered significant global interest since the early 1970s. Although the present Mars is considered hostile to life, several missions have taken place on its surface [59]. The Viking mission found no evidence of life, likely due to the scarce sensitivity of the GC-MS instrument [26,60,61]. In contrast, the Mars Science Laboratory onboard the Curiosity rover detected organic compounds, methane concentrations in the Martian atmosphere, and reduced sulfur compounds [62,63]. More recently, the discovery of hydrated salts at Recurring Slope Lineae (RSL) has suggested the possible presence of liquid water [64], further increasing the plausibility of finding life or its traces on Mars.

Missions on Mars have transitioned from directly searching for traces of life to employing indirect approaches aimed at identifying the planet’s surface as a potentially habitable environment (e.g., searching for water or essential elements). However, the focus has now returned to the search for life or its traces [65]. Indeed, new generation life detection programs are currently ongoing. NASA Mars 2020 successfully landed the Perseverance rover on the Martian surface in February 2021, which is set to return rock and soil samples by 2033. Meanwhile, the ESA ExoMars Rosalind Franklin rover, slated to be launched in the near future, will conduct soil analyses up to 2 m depth. Clearly, in order to detect potential traces of life beyond Earth from these missions, it is crucial to focus on indicators of life that are of biological origin, such as nucleic acids. This assumption, however, relies on the search for life being closely tied to terrestrial life forms. At the moment, however, no mission specifically targets DNA search. There are still several factors to address in supporting DNA research on the Martian surface. DNA is highly sensitive to various chemical and physical conditions, such as the planet’s intense radiation environment and the presence of minerals, which have different mechanisms for adsorbing biomolecules, depending on the chemical properties of the molecules and the types of minerals involved.

The PCR amplification method has proven to be the most successful life detection and classification tool developed to date. The PCR method on Earth is largely based on targeting highly conserved genetic regions, such as those within the 16S and 23S ribosomal RNA genes [66]. Since this amplification technique has also been proposed for in situ life detection experiments [31,47,67], we tested its ability to recover and amplify fungal DNA after exposure to Fe ions, either alone or mixed with a Martian regolith simulant (S-MRS). For this experiment, fungal DNA was extracted from the black fungus *C. antarcticus*, which, due to its polyextremophilic nature, is a very resilient microorganism and has been widely used as a test model for astrobiological studies [50]. Its resistance can be utilized to assess the stability and detectability of microbial biomolecules under simulated conditions (e.g., radiation, desiccation, oxidizing species) [56,68,69,70]. However, differences in adaptation mechanisms between terrestrial life forms and hypothetical extraterrestrial organisms should be taken into account.

In this study, to assess the effects of irradiation and minerals on DNA and on its detection, we used PCR and quantitative PCR assays, amplifying various gene lengths, to gain a more exhaustive assessment of potential damage to the fungal genome. Initially, we successfully resuspended and quantified all the DNA samples after exposure to Fe ions (Table S1).

Our results demonstrated successful amplification for the ITS region (700 bp) in all the exposed samples (Figure S1A). However, it is evident that the PCR bands of amplified DNA mixed with S-MRS are less intense compared to directly exposed DNA (Figure S1A). These results were supported by the light intensity measurements, that showed lower values for the amplified DNA samples mixed with S-MRS analogue, while no significant differences in intensity were reported among the directly exposed DNA samples even at the highest dose (Figure S1B). In contrast, the amplification of the 1600 bp-length region showed no amplicons for any of the DNA samples mixed with S-MRS (Figure S2A), revealing a noticeable decrease in light intensity with the increase in radiation dose (Figure S2A,B). A similar pattern was observed in the whole-genome amplification using RAPD (Figure S3A), where no amplification or light intensity was detected in the DNA samples mixed with the S-MRS analogue (Figure S3A,B).

Quantitative PCR results revealed high amplification values for all the directly exposed samples, with a decrease in amplified copy numbers for samples exposed to 1000 Gy. No amplification was observed in all DNA samples mixed with S-MRS analogue (Figure 2A). These results are in accordance with [71], where the authors reported that the presence of Martian regolith simulants does not provide protection but instead increases the production of secondary radiation, reducing the likelihood of dormant fungal cells surviving in the Martian subsurface. Given the shielding depth of the material (S-MRS = 1.53 g/cm^3^) and the parameters set for the accelerated ions (energy = 500 MeV/n, LET in water = 200 keV/μm), we can rule out that these results are due to the positioning of the DNA within the Bragg peak region along the trajectory of the particles. The amplification data of directly exposed DNA were also useful to calculate DNA lesions applying the Poisson distribution. At the highest dose (1000 Gy), a low rate of ~0.11 lesions/0.9 kb was reported (Figure 2B; Table 2). Although good amplification values were obtained, it is important to consider the decrease in amplification of directly exposed DNA when compared to the exposure of fungal cells. Indeed, ref. [71] reported amplification values approximately 10 times higher when *C. antarcticus* colonies were directly exposed or mixed with S-MRS analogue to the same doses of Fe ions. This discrepancy can be attributed to the protective role of the cellular structure. For instance, biofilms, which are an organized aggregate of microorganisms living within a self-produced extracellular polymeric substance (EPS) matrix, can enhance microbial resistance to UV radiation [72], extreme temperature and pH [73,74], high salinity [75], high pressure [76], poor nutrients [77], among other stressors.

Our findings suggest that the Martian regolith may pose significant challenges to DNA preservation and detection due to potential degradation and adsorption effects. The lack of amplification in DNA samples mixed with S-MRS may be attributed to interference from mineral interference during PCR and quantitative PCR reactions, although all mineral residues were carefully removed during resuspension. It has been largely reported that the high concentration of metals, clay, or silicates in Martian regolith analogues can lead to DNA degradation or DNA binding to the soil [78].

In this context, the composition of minerals should be taken into account. As widely reported, clay minerals, due to their surface electrostatic properties, have a strong ability to polarize molecules like DNA and RNA [45,79]. This strong absorption capacity allows clay minerals to protect against enzymatic digestion [80], UV radiation [81], and X-rays [82]. Other minerals, such as jarosite and the silica-bearing minerals olivine, diopside, labradorite, and apatite, also adsorb DNA. However, DNA has a low affinity for silica, though its adsorption can be enhanced by the presence of cations [83]. The study of [45] described the recovery of DNA spike from various minerals, showing lower recovery from clay minerals, and higher recovery from silica minerals (plagioclase, olivine, pyroxene) and iron-rich minerals like iron oxides (magnetite and hematite) and iron oxyhydroxides (goethite and ferrihydrite). In our study, the S-MRS analogue primarily consists of silica minerals, iron oxides, and quartz. Despite these minerals having lower DNA-binding affinity, we observed no amplification in DNA samples mixed with S-MRS analogue. On the contrary, a recent experiment by [46] reported successful DNA recovery from JSC MARS-1 analogue, which shares a similar composition with S-MRS. Additionally, further experiments using different regolith analogues (e.g., MGS-1S, JSC MARS-1) and different types of radiation (i.e., under Galactic Cosmic Rays simulation, see [84]) will be performed to fully elucidate the preservation of DNA into minerals and other oxidant species (e.g., perchlorates).

To conclude, a major challenge for astrobiologists is developing strategies to enhance the possibility of finding life beyond Earth, with a key approach being the study of terrestrial life, as it remains our only known example of life. One of the most effective approaches is to base research on the terrestrial. Nucleic acids, particularly DNA, are central to this effort, aligning with the hypothesis that life on Earth and potential past or present life on Mars could share fundamental building blocks.

Currently, no Mars rover is equipped to directly detect DNA. However, rovers equipped with prototype instruments are being tested for biomolecule detection [85].

To conclude, our experiments have shown that highly sensitive molecular biology techniques, like PCR and qPCR, can be adapted for detecting life on Mars, even after exposure to heavy ion irradiation. Our study confirmed that these methods can amplify DNA, but they are not sensitive enough to detect fragmented DNA caused by factors like minerals and radiation. This has direct implications for planning future Mars missions, indicating that life detection experiments should incorporate optimized DNA extraction methods and employ complementary techniques, such as spectroscopy or mass spectrometry, to detect degraded biomolecules. Additionally, understanding regolith interactions with DNA helps refine strategies for sample collection and analysis in upcoming missions, as well as improve the analysis of Martian samples, particularly those returned with the Mars Sample Return (MSR) mission.

## 5. Conclusions

The results of this study reported the difficulty of amplifying DNA from a Martian regolith simulant, highlighting important implications for the search for life on Mars. These findings suggest that a regolith may contain components that inhibit DNA extraction or amplification with available methods, such as reactive minerals or adsorption onto mineral surfaces. This could pose a significant challenge for in situ life detection missions. However, this result does not entirely rule out the possibility of detecting biomolecules on Mars. It highlights the need for alternative extraction methods and more robust amplification techniques, such as next-generation sequencing coupled with spectroscopic analyses. These technologies should be designed to minimize interference from the environmental conditions (e.g., radiation, minerals, and oxidants). Additionally, it underscores the importance of testing and optimizing protocols using Mars-relevant analogues to improve the likelihood of detecting potential biosignatures in future missions. Moreover, understanding how different minerals affect biomolecule preservation may support future sample collection strategies, helping also to identify the most promising sites for life detection.

The experimental conditions explored in this study have direct relevance to the lithopanspermia hypothesis, which posits the theoretical possibility of life being transferred, embedded within rock fragments ejected by impact events, between early Mars and Earth during the Late Heavy Bombardment period 29].

## Figures and Tables

**Table 1:** DNA amounts of resuspended samples after irradiation exposure. The resuspended DNA from all the samples was diluted to the final concentration of 0.1 ng/mL. S-MRS = Sulfatic Mars Regolith Simulant.

Experimental Conditions	DNA Concentration (ng/μL)
Ctr	7.2
50 Gy	7.75
50 Gy S-MRS	0.03
250 Gy	6.82
250 Gy S-MRS	0.01
500 Gy	4.84
500 Gy S-MRS	0.01
1000 Gy	7.69
1000 Gy S-MRS	0.03

**Table 2:** Representation of raw fluorescence values obtained after real-time PCR amplification of the LSU gene of DNA extracted from samples exposed to increased doses of iron ions.

Samples	Mean	Final Read	Relative Amplification	Lesion Frequency	Lesion/0.9 kb
Ctr	23.00	−16.50	1	0	0
50 Gy	23.50	−16.00	0.97	0.03	0.03
250 Gy	23.00	−16.50	1	0	0
500 Gy	23.50	−16.00	0.97	0.03	0.03
1000 Gy	25.00	−14.50	0.88	0.13	0.12

## Data Availability

The original contributions presented in this study are included in the article/Supplementary Materials. Further inquiries can be directed to the corresponding author.

## Supplementary Materials

The following supporting information can be downloaded at https://www.mdpi.com/article/10.3390/life15050716/s1, Figure S1: (A) Agarose gel electrophoresis of PCR amplification products of the ITS region (700 bp) of *C. antarcticus* extracted DNA, exposed to increase doses of accelerated iron ions (500 MeV/n). Ctr: DNA extracted from *C. antarcticus* colonies not exposed to radiation treatment. (B) Relative light intensity values obtained with Image J software from ITS bands. Light intensity values from each treated sample are normalized with respect to the control.; Figure S2: (A) Agarose gel electrophoresis of PCR amplification products of the ITS-LSU region (1600 bp) of *C. antarcticus* extracted DNA, exposed to increase doses of accelerated iron ions (500 MeV/n). Ctr: DNA extracted from *C. antarcticus* colonies not exposed to radiation treatment. (B) Relative light intensity values obtained with Image J software from LSU bands. Light intensity values from each treated sample are normalized with respect to the control. Figure S3: (A) Agarose gel electrophoresis of PCR whole genome (RAPD assay) amplification of *C. antarcticus* extracted DNA, exposed to increase doses of accelerated iron ions (500 MeV/n). Ctr: DNA extracted from *C. antarcticus* colonies not exposed to radiation treatment. (B) Relative light intensity values obtained with Image J software from RAPD bands. Light intensity values from each treated sample are normalized with respect to the control.
