# Supplementary material for: Exploring Life Detection on Mars: Understanding Challenges in DNA Amplification in Martian Regolith Analogue After Fe Ion Irradiation
Source: Life (Basel). 2025 Apr 29;15(5):716. doi: 10.3390/life15050716 (PMC12113113; doi:10.3390/life15050716)
Supplement: Supplementary file 1 [file life-15-00716-s001.zip › life-3531523-supplementary.pdf]

Supplementary Materials

Exploring Life Detection on Mars: Understanding Challenges in DNA Amplification in Martian Regolith Analogue After Fe Ion Irradiation

Alessia Cassaro [1,2], Claudia Pacelli [1,2,*] and Silvano Onofri [2]

[1] Science and Innovation Directorate, Italian Space Agency, via del Politecnico, 00133 Rome, Italy; alessia.cassaro@asi.it
[2] Department of Ecological and Biological Sciences, University of Tuscia, Largo dell'Università snc, 01100 Viterbo, Italy; onofri@unitus.it
* Correspondence: claudia.pacelli@asi.it; Tel.: +39-068567466

Single Gene PCR and Random Amplified Polymorphic DNA Analysis

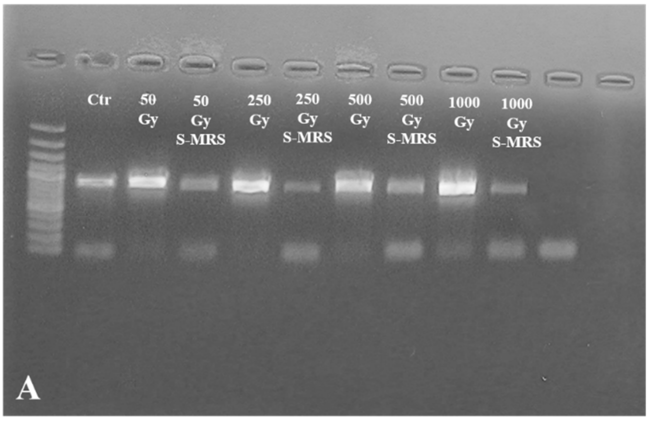

Samples	Light intensity
Ctr	1
50	2.95
50 S-MRS	0.68
250	3.39
250 S-MRS	0.27
500	3.22
500 S-MRS	2.84
1000	3.95
1000 S-MRS	0.38

B

Figure S1. A) Agarose gel electrophoresis of PCR amplification products of the ITS region (700 bp) of *C. antarcticus* extracted DNA, exposed to increase doses of accelerated iron ions (500 MeV/n). Ctr: DNA extracted from *C. antarcticus* colonies not exposed to radiation treatment. **B)** Relative light intensity values obtained with Image J software from ITS bands. Light intensity values from each treated sample are normalized with respect to the control.

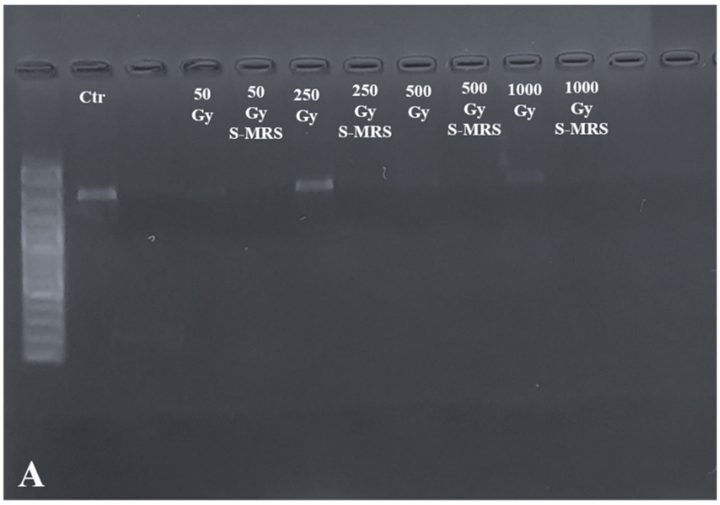

B

Samples	Light intensity
Ctr	1
50	0.04
50 S-MRS	0
250	0.97
250 S-MRS	0
500	0.33
500 S-MRS	0
1000	0.33
1000 S-MRS	0

Figure S2. A) Agarose gel electrophoresis of PCR amplification products of the ITS-LSU region (1600 bp) of *C. antarcticus* extracted DNA, exposed to increase doses of accelerated iron ions (500 MeV/n). Ctr: DNA extracted from *C. antarcticus* colonies not exposed to radiation treatment. **B)** Relative light intensity values obtained with Image J software from LSU bands. Light intensity values from each treated sample are normalized with respect to the control.

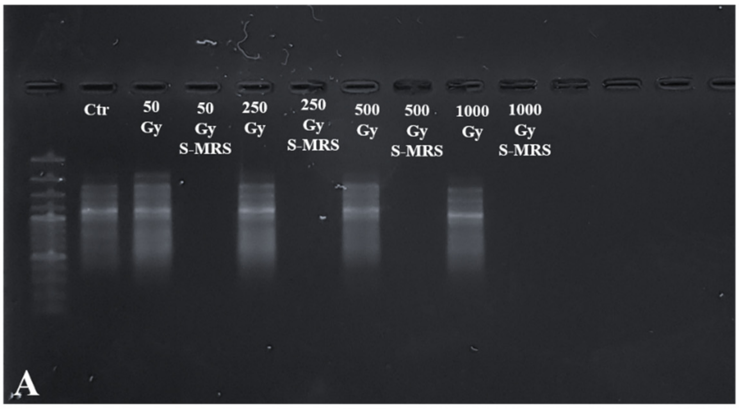

B

Samples	Light intensity
Ctr	1
50	4.58
50 S-MRS	0.02
250	3.39
250 S-MRS	0.04
500	2.70
500 S-MRS	0
1000	2.26
1000 S-MRS	0

Figure S3. A) Agarose gel electrophoresis of PCR whole genome (RAPD assay) amplification of *C. antarcticus* extracted DNA, exposed to increase doses of accelerated iron ions (500 MeV/n). Ctr: DNA extracted from *C. antarcticus* colonies not exposed to radiation treatment. **B)** Relative light intensity values obtained with Image J software from RAPD bands. Light intensity values from each treated sample are normalized with respect to the control.
